# Clinical picture and long-term symptoms of SARS-CoV-2 infection in an Italian pediatric population

**DOI:** 10.1186/s13052-022-01270-1

**Published:** 2022-05-21

**Authors:** Silvia Bloise, Sara Isoldi, Alessia Marcellino, Enrica De Luca, Anna Dilillo, Saverio Mallardo, Vanessa Martucci, Mariateresa Sanseviero, Emanuela Del Giudice, Donatella Iorfida, Rita Leone, Alessia Testa, Beatrice Frasacco, Pietro Gizzone, Claudia Proietti Ciolli, Alessandro Sinceri, Francesca Zuliani, Elena Zanardi, Anna Gambarotto, Anna Lisa Grandinetti, Flavia Ventriglia, Riccardo Lubrano

**Affiliations:** 1 Dipartimento Materno Infantile E Di Scienze Urologiche, Sapienza Università Di Roma, UOC Di Pediatria e NeonatologiaOspedale Santa Maria Goretti, Polo Pontino, Rome, Lazio Italy; 2 Direzione Sanitaria Azienda, Sanitaria Locale Di Latina, Latina, Lazio Italy

**Keywords:** Long COVID, Children, Risk factors, Psychological effects

## Abstract

**Background:**

SARS-CoV-2 infection in the pediatric age group has a milder course than in adults, but in some cases even children may present with severe forms or develop long-term consequences. The aim of this study was to analyze the clinical features, long-term effects, lifestyle changes and psychological effects of SARS-CoV-2 infection in a pediatric sample of the Italian population.

**Methods:**

We conducted a telephone survey among 3075 children infected with SARS-CoV-2 in the Latina Local Health Authority. Outcomes included: clinical features of infection, long-term symptoms, lifestyle changes and emotional symptoms during the illness. The information obtained was automatically linked to a spreadsheet and analyzed.

**Results:**

One thousand four hundred thirteen children agreed to participate in the study; the mean age was 112.8 ± 21.9 months. Children were infected mainly inside familial clusters (59.6%; *n* = 842); 99% (*n* = 1399) of children were asymptomatic or exhibited mild symptoms. 20% (*n* = 259) of children experienced long-term symptoms; risk factors were: older age, higher body mass index and longer duration of infection.

Throughout the period of infection, children spent most of the time on devices like tv-video, social media and mobile phone for non-educational activities. 58.8% (*n* = 620) of parents expressed a negative opinion about distance learning. Finally, we observed that 49,6% (*n* = 532) of children experienced psychological symptoms during quarantine period.

**Conclusion:**

Despite a lower susceptibility to COVID-19 in children, it is important to keep the focus high in children, both because of the possible long symptoms after infection and the impact on a children’s mental and physical health due to pandemic. We believe that the return to school or other extracurricular activities are important to correct some of the risk factors for the long COVID syndrome, as obesity, and to limit the cultural damage generated by distance learning and psychological effects related to restrictive measures.

## Background

The 2019 novel coronavirus disease (COVID-19) was officially declared as a public health emergency of international concern in March 2020 [1]. Despite the implementation of preventive strategies to contain the infection, millions of people were infected by SARS-CoV-2 and millions of deaths have been recorded all over the world [2].

Most of the early published studies focused mainly on the characteristics of the disease in the adult and elderly population [3, 4], considered to be at the highest risk of severe disease and death [5]. In fact, since the beginning of the outbreak, children are seemed to have an asymptomatic or a milder clinical course compared to adults [6–8] and the focus on them has been initially lower. Subsequently, however, numerous reports have been conducted to describe the clinical features of the infection also in pediatric age [9], the secrets of their immune response [10] and the differences with adult population [11, 12]. Researches have, also, focused the attention on the possible negative effects on mental health symptoms due to rigid measures implemented during lock down to contain the spread of the infection [13, 14].

Recently, although increasing attention has been given in adult patients to the long-term effects of COVID-19, generating another category of patients called “Long Covid” [15, 16], few data have been collected in the pediatric population [17, 18].

Therefore, we wanted to conduct a survey among all children infected with SARS-CoV-2 in Latina.

The primary aim of our study was to evaluate long-term symptoms of infection, lifestyle changes and emotional and psychological aspects of a sample of Italian children infected by SARS-CoV-2; Secondary aim was to investigate the demographic characteristics and the clinical features of infection of these children and their parents.

## Methods

Following the activation of a pediatric outpatient clinic post-COVID in our hospital, we have taken charge of all children infected by SARS-CoV-2 between March 2020 and March 2021 and living at Latina Local Health Authority (ASL of Latina).

Institutional review board of Maternal and Child Health Department of the Local Health Authority of Latina approved the study protocol (protocol 02–15/03/2021).

We developed a survey with a web-form link to collect data (Google forms). The survey was conducted through a questionnaire administered by telephone by the pediatricians assigned to the management of outpatient clinic post-COVID. All families with children infected by SARS-CoV-2 between March 2020 to March 2021, were invited to participate to the survey. The questionnaire included specific questions for parent and children for participants ≥ 6 years, while only the parent’s section was required for younger kids. In particular, the questionnaire included different items about children’s demographic characteristics, clinical features of infection, long-term symptoms of infection, lifestyle changes and the main activities practiced during lock down and a final section dedicated to emotional and psychological aspects; Furthermore, the questionnaire included also a section that investigated parents’s demographic characteristics and clinical features and the course of infection. A verbal informed consent was obtained before beginning the interview. The information obtained was automatically linked to a spreadsheet and analyzed.

In relation to the post-COVID pediatric outpatient clinic of our hospital, the local protocol implemented includes: clinical examination with detailed history of previous infection and the possible presence of current symptoms, blood tests, evaluation of glomerul and tubular function and proteinuria on 24 h urine sample, electrocardiogram, spirometry, multidimensional fatigue questionnaire (Peds-QL Fatigue). Then, depending on the symptoms that emerged during the first clinical examination, our children are referred to specialist evaluations: cardiological, otolaryngology, dermatological, pneumology.

### Patient’s characteristics

Demographic data collected included: sex, educational level and job of parents; sex, blood group, comorbidity, weight and height of children.

### Infection-related characteristics

The infection-related characteristics collected for both children and parents were: place of infection (family, school, hospital, sporting activity, unknown); index case; contagions in the family; duration of infection; symptoms of infection in children and their parents; complications; hospitalization; therapies; referring physician, the performance of serological tests and result.

### Long-term characteristics of infection

The characteristics of infection analyzed were: long-term symptoms of infection; investigations performed for these symptoms, therapies and outcome.

### Lifestyles changes

Lifestyle changes were collected by asking parents about their children's most frequent activities during lockdown; hours spent studying during the day or hours spent using social media, gaming with a notebook, smartphone or other devices or watching television correlating with the age group [Group A: 72–120 months (elementary school); Group B 121–156 months (middle school); Group C:157–216 months (high school)].

### Emotional and psychological effects

This section was addressed only for children ≥ 6 years. We asked them how worried they felt about COVID-19 (using a scale of 1 to 3), what worried them the most (transmitting or contracting the infection), what they missed the most between the following activities (attend school, hang out with friends, play sports or other activities) and if they had exhibited any of these symptoms since the start of the pandemic (difficulty in falling asleep, nocturnal awakenings, insomnia, anxiety, apathy, difficulty in concentrating, depression, sense of loneliness).

### Statistical analysis

The statistical analysis was performed with JMP® 16.1.0 program for Mac (SAS Institute Inc). The qualitative variables were described as the distribution of absolute frequencies and percentages. We used Fisher’s test to compare categorical variables. For the data expressed as continuous variables the approximation to normal of the distribution of the population was tested with the Shapiro–Wilk and Anderson Darling test. As results were asymmetrically distributed, data are expressed as median and 25th and 75th quartile, and for analysis non-parametric tests were used. We used the Kruskal–Wallis nonparametric one-way analysis of variance to examine the changes of each parameter between the subgroups: the null hypothesis was that the groups for the same parameter all came from the same distribution. When the Kruskal–Wallis test was significant, we used the Wilcoxon test to compare the intragroup differences. A *p* value < 0.05 was considered significant.

## Results

We contacted the family of 3075 children (5.7% of all children aged 0–18 years living in the ASL of Latina) who were infected with SARS-CoV-2 between March 2020 and March 2021, to invite them to come to our pediatric post-COVID outpatient clinic for a visit. Initial work consisted of a telephone interview, conducted by pediatricians assigned to manage the post COVID outpatient clinic. 10.8% (*n* = 333) did not answer the telephone and 43.2% (*n* = 1329) declined to participate; 46% (*n* = 1413) agreed to participate to the survey and were finally enrolled in the study.

The clinical and demographic characteristics are summarized in Table 1.

**Table 1:** Clinical and demographic characteristics of subjects included in the study (parents and children)

**Children’s characteristics**		
Sex (F/M), % (n)	48.8% (689) / 51.2% (723)	
Age (median 25°—75° quartile), years	10 (6 – 13)	
Blood group % (n)	Unknown:	49.3% (696)
	**A + :**	**17.3% (244)**
	A -:	1.7% (24)
	B + :	5.5% (78)
	B -:	0.8% (11)
	AB + :	3.0% (43)
	AB -:	0.5% (3)
	**0 + :**	**17.5% (247)**
	0 -:	4.5% (64)
Comorbidities % (n)	None:	76.8% (1084)
	**Allergies:**	**15.3% (216)**
	Respiratory diseases:	2.5% (36)
	Cardiac diseases:	1.6% (22)
	Gastroenterological diseases:	1.6% (23)
	Neurological diseases:	2.4% (34)
	Nephrological diseases:	0.5% (7)
	Endocrinological diseases:	1.4% (20)
	Genetic diseases:	1% (14)
	Immunodeficiencies:	0.1% (2)
	Rheumatological diseases:	0.4 (5%)
Weight (mean ± SD), Kg	38.5 (22.5 – 54.25)	
Height (mean ± SD), cm	141 (116 – 162)	
**Parent’s characteristics**		
Educational level % (n)	**Mother**	
	Elementary schools:	1.4% (20)
	Middle schools:	20.8% (294)
	High schools:	54.4% (768)
	Degree:	23.4% (330)
	**Father**	
	Elementary schools:	2.1% (29)
	Middle schools:	27.2% (384)
	High schools:	52.9% (747)
	Degree:	17.8% (252)
Type of job % (n)	**Mother**	
	Housewife:	33.8% (477)
	Teacher:	7.9% (112)
	Doctor/nurse/health worker:	5.6% (79)
	Employee:	22.4% (316)
	Worker:	6% (85)
	Freelancer:	6.5% (92)
	Merchant:	4.1% (58)
	Unemployed:	6.1% (86)
	**Father:**	
	Teacher:	1.4% (20)
	Doctor/nurse/health worker:	2.2% (31)
	Employee:	23.3% (329)
	Worker:	27.1 (383)
	Freelancer:	15.1% (213)
	Merchant:	5.9% (84)
	Unemployed:	4.3% (61)
	Military:	3.4% (48)
	Police man:	0.8% (8)

It is interesting to note that, although 49% (*n* = 696) of the parents of infected children was not aware of the blood group of their children, in the remaining cases, group A + prevailed in 17.3% (*n* = 244) of cases and group 0 + in 17.5% (*n* = 247).

### Infection-related characteristics

**Fig. 1:**
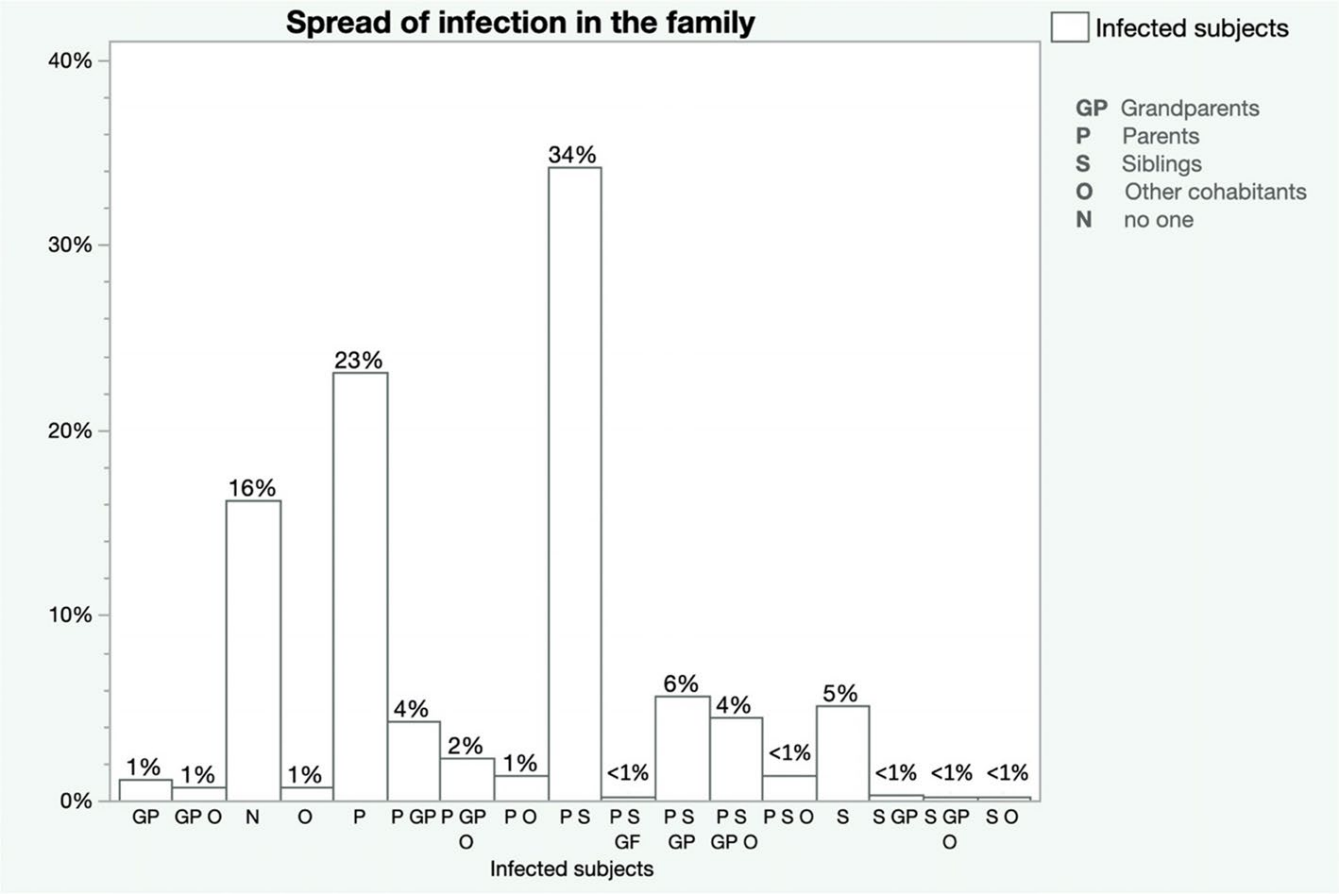
Spread of infection in the family of the infected children

Children were infected mainly inside familial clusters (59.6%; *n* = 842), secondly at school (21.5%; *n* = 304), during sporting activity (1.9%; *n* = 27) and in hospital (0.6%; *n* = 8). In 16,4% (*n* = 232) of cases there was not possible track the contagion. Index case was represented mainly by parents (65.3%, *n* = 543), siblings (17.8%; *n* = 148), grandparents (12.4%, *n* = 103) and other second-degree relatives (8.4%, *n* = 71). In fact, family clusters involved parents and siblings in most cases, and only in 16% of cases did the infection represent an isolated event within the family (Fig. 1).

During infection 27.5% (*n* = 388) of children were asymptomatic, while the remaining cases showed mild symptoms.

The most common symptoms in children were: fever (38.8%,*n* = 549) with a duration of 1 (1 – 2) days and temperature of 38 (37.5 – 38.5) °C, headache (24.1%,*n* = 340), rhinitis (21.7%,*n* = 306), asthenia (20%, *n* = 283), anosmia (15.7%,*n* = 222) and ageusia (15.2%,*n* = 214), arthromyalgia (13.7%,*n* = 193), cough (13.4%,*n* = 189), pharyngodynia (8.8%, *n* = 124), inappetence (7.4%,*n* = 105), gastrointestinal symptoms (3.6%,*n* = 37), conjunctivitis (2.3%,*n* = 33), and other symptoms (1.7%, *n* = 17).

In parents, we observed, fever (mothers 58.3% (*n* = 577), fathers 61.7% (*n* = 510)), headache (mothers. 51.8% (*n* = 512), fathers 41.1% (*n* = 340)), asthenia (mothers. 51.2% (*n* = 506), fathers 48% (*n* = 397)), arthromyalgia (mothers. 58.3% (*n* = 577), fathers 54.2% (*n* = 448)), rhinitis and cough (mothers 47.2% (*n* = 390), fathers 51.6% (*n* = 510)), pneumonia (mothers 8.1% (*n* = 80), fathers 11.6% (*n* = 96)) of these 42.4% of mothers and 53.1% of fathers were hospitalized, anosmia (mothers 59.7% (*n* = 590), fathers 46.7% (*n* = 386)) and ageusia (mothers 60.1% (*n* = 594), fathers 48.1% (*n* = 398)).

**Fig. 2:**
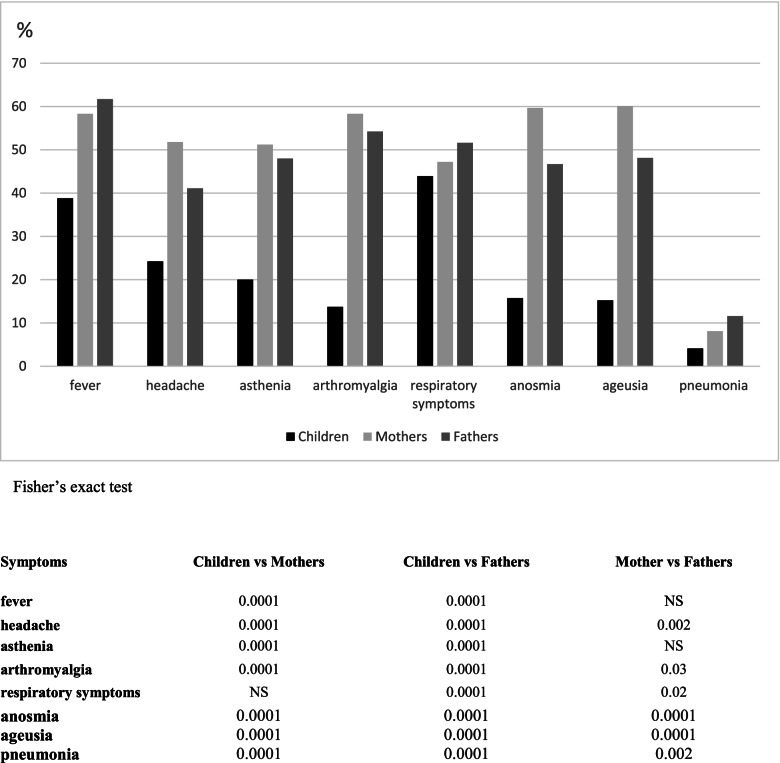
Clinical symptoms in children and their parents during SARS-CoV-2 infection

As shown in Fig. 2, we observed a significant lower incidence of all clinical manifestations in children compared to parents, excepting for respiratory symptoms where the incidence was significantly higher only in fathers, whereas mothers and children showed no significant differences. Among parents, we observed that anosmia, ageusia and headaches were more frequent in women, while pneumonia prevailed in men.

Ninety Nine percent (*n* = 1399) of the children were cared at home, mainly by family pediatricians (69.8%, *n* = 986) or general practitioners (26.8%,*n* = 379), or territorial ASl doctors (3.3%,*n* = 46%), while 1% (*n* = 14) needed hospitalization. Patients who were hospitalized showed: pneumonia in 3 cases, pericarditis with pericardial effusion in 1 and Multisystem Inflammatory Syndrome (MIS-C) in 1.

Of children treated at home, 51.6% (*n* = 728) did not take drugs, 29.8% (*n* = 422) took acetaminophene, 23.3% (*n* = 326) supplements as Vitamins, 12.7% (*n* = 180) antibiotics, 5.7% (*n* = 83) corticosteroids and 4% (*n* = 58) other anti-inflammatories drugs.

The mean time length necessary to obtain the negativization of nasopharyngeal molecular swab tests was 18 (13 – 24) days. After 3 months from the infection 6.4% (*n* = 90) of the children perform the serological test for anti-SARS-CoV-2 IgM and IgG, with positive result in 83.3% (*n* = 75). In addition, after negativization of nasopharyngeal swab, 13.8%% (*n* = 195) of the children had contact with infected individuals, of these 58.4% (*n* = 114) repeated the molecular swab, which was negative in all cases examined.

### Long-term effects of COVID infection

Patients were assessed on average 87.49 ± 56.44 days after COVID-19 diagnosis.

After the infection, 80% (*n* = 1155) of children had not persistent symptoms characteristic of long COVID, whereas 20% (*n* = 258) had experienced persistent symptoms, of these 160 (62%) were females.

Comparing children with persistent symptoms (PSC: children with persistent symptoms *n* = 258) and children who did not experience these complications (HC Heathy children), we showed that children with persistent symptoms were older [PSC 140 (89—173) vs HC114 (67—155) months *p* < 0.000], had a longer duration of SARS-CoV-2 infection [PSC 20 (14—28) vs HC 18 (12—24) days *p* < 0.0004] and higher body max index (BMI) [PSC 19.52 (16.33 – 21.77) vs HC 18.73 (16.33 – 21.77) days *p* < 0.006] than children without persistent symptoms (Table 2).

**Table 2:** Comparison children with persistent symptoms (PSC: children with persistent symptoms *n* = 259) and children who did not experience these complications (HC Heathy children). The data were expressed as median, 25th and 75th quartile and as medians ± SD or in percentage

	**Children with persisting symptoms** (PSC)	**Healthy children** (HC)	***p* value**
**Age** *(median 25°- 75° quartile) months*	140 (89—173)	114 (67—155)	*p* < .0001
**Sex** *(F/M), % (n)*	62% (160) / 38% (98)	45.9% (530)/54.1% (625)	NS
**Nationality** *% (n)*	Italian 100% (258)	Italian 99.1% (1145) Indian 0.9% (10)	NS
**BMI** *(median 25°- 75°quartile), Kg/m2*	19.52 (16.33 – 21.77)	18.73 (16.33 – 21.77)	*p* < .006
**Duration of infection** *(median 25°- 75°quartile), days*	20 (14—28)	18 (12—24)	*p* < .0004
**Severity of infection** *% (n)*			NS
Asymptomatic	10.9% (28)	31.1% (360)	
Mild	89.1% (230)	68.9% (795)	
**Follow-up** *(mean* ± *SD), days*	87.49 ± 56.44	87.49 ± 56.44	NS

*NS* not significant

**Fig. 3:**
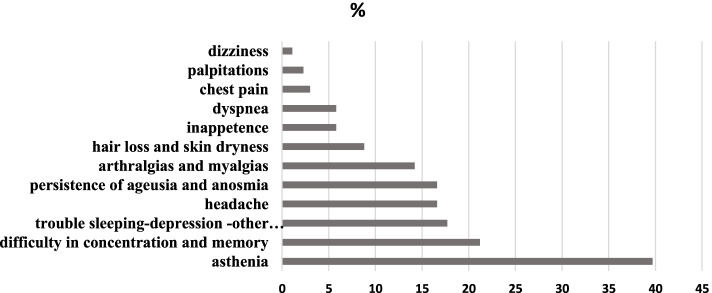
Symptoms experienced after SARS-CoV-2 infection in children

The most common symptoms were asthenia (39.9%, *n* = 103), difficulty in concentration and memory (21.3%, *n* = 55), trouble sleeping-depression and other neuropsychiatric disorders (17.8%, *n* = 46), headache (16.7%, *n* = 43), persistence of ageusia and anosmia (16.7%, *n* = 43), arthralgias and myalgias (14.3%, *n* = 37). Detailed graphical representation of symptoms experienced after SARS-CoV-2 infection is shown in Fig. 3.

Of patients that have experienced symptoms after infection, 89.5% (*n* = 231) did not perform investigations for these symptoms, while 10.5% (*n* = 27) performed investigations (blood tests, instrumental tests or specialized visits) with negative results in 56% (*n* = 15) and positive in 44% (*n* = 12) of cases; the results of these investigations were pericardial effusion (3 cases),, hypertension (1 case), anxiety disorder (3 cases), tachyarrhythmia (1 case), depression (1 case), asthmatic bronchitis (2 cases), dermatitis (1 case),.

74.4% (*n* = 193) of patients did not take any medications for these symptoms; 18% (*n* = 47) took supplements (vitamins and probiotics), 6.5% (*n* = 17) paracetamol, 3.7% (*n* = 10) antibiotics and 1.6% (*n* = 4) anti-inflammatories drugs.

### Lifestyles changes

The most frequent activities practiced throughout the period of infection were: tv-video and web meeting for non-educational activities (56.5%, *n* = 617), tv-video and web meeting for educational activities, mainly school (49.5%, *n* = 542), cell phone for games, video and chat (28.6%, *n* = 316), computer gaming (20.3%, *n* = 222) and physical activity only in the 14.2% (*n* = 156) of the children.

In this period, 12.3% of parents noticed an overeating among in their children and an increase in sleeping time (especially in the afternoon). In parallel, we found an increase in body weight in 30.6% (*n* = 432) of the children, while in 7,5% (*n* = 106) a condition of loss of appetite. Instead, 61.9% (*n* = 875) of children remained stable in weight.

Table 3 shows the hours that children spent on study and leisure time in the three age groups of our survey: [Group A: 72–120 months (elementary school, *n* = 386); Group B 121–156 months (middle school, *n* = 298); Group C:157–216 months (high school *n* = 376)]. In all groups we recorded that most of the hours were used for leisure, while the hours used for study gradually increased with age and education level. Finally, the hours spent using social media, playing with a notebook, smartphone or other devices also increased significantly in group B and C compared to group A, while there were no differences between group B and C.

**Table 3:** Leisure hours and hours for study in the three different age groups, Group A: 72–120 months (elementary school); Group B 121–156 months (middle school); Group C:157–216 months (high school). All data were expressed as median, 25th and 75th quartile

	**Hours for study**	**Leisure hours**
**Group A** (*n* = 386)	2 (2–3)	4 (3–5)
**Group B** (*n* = 298)	3 (2–5)	5 (3–6)
**Group C** (*n* = 376)	4 (2–6)	5 (3–6)

**Hours for study**

Kruskal–Wallis *p* < .001

Wilcoxon test: group A vs group B *p* < .0001; group A vs group C *p* < .0001; group B vs group C *p* < .0001

**Leisure hours**

Kruskal–Wallis *p* < .001

Wilcoxon test: group A vs group B *p* < .0001; group A vs group C *p* < .0001; group B vs group C *p* < .06

**Hours for study vs Leisure hours**

Wilcoxon test: group A: *p* < .0001; group B: *p* < .0001: group C: *p* < .0001

Regarding distance learning, 94.7% (*n* = 1020) of parents reported that their children were able to follow this new method of teaching, in particular: 65.1% (*n* = 686) alone, 34.7% (*n* = 365) with the help of parents, 2% (*n* = 21) with the help of older brothers, 0.8% (*n* = 8) with grandparents, 0.6% (*n* = 6) with other people.

However, 58.8%(*n* = 620) of parents think distance learning has negatively affected their children's academic learning, for 20.9% (*n* = 221) significantly and for 37.9%(*n* = 399) moderately, while 41.2% think that distance learning did not alter learning (*n* = 434).

### Emotional and psychological effects

**Fig. 4:**
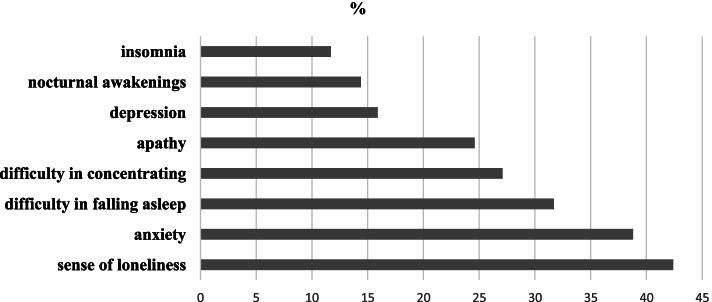
Prevalence of psychological symptoms in children older than 6 years during the period of illness

Children older than 6 years reported their mood during the period of illness as always good (50.5%, *n* = 541), fluctuating (42.2%, *n* = 453), depressed (7.4%, *n* = 79). In particular, they reported the following psychological symptoms: difficulty in falling asleep, nocturnal awakenings, insomnia, anxiety, apathy, difficulty in concentrating, depression, sense of loneliness. The percentages at which these symptoms occurred were: sense of loneliness (42,3%, *n* = 203), anxiety (39%, *n* = 187), difficulty in falling asleep (31,7%, *n* = 152), difficulty in concentring (27,3%, *n* = 131), apathy (24,6%, *n* = 118), depression (15,8%, *n* = 76), nocturnal awakenings (14,6%, *n* = 70), insomnia (11,7%, *n* = 56) (Fig. 4).

When we asked to the children what they missed the most, 45.8% (*n* = 491) responded going out with friends, 22.7% (*n* = 244) attending school, 22% (*n* = 236) sports activities, 4,9% (*n* = 53) nothing and 4.6% (*n* = 49) other activies.

## Discussion

Our findings showed that 20% of children infected with SARS-CoV-2 are at risk of experiencing persistent symptoms after the infection. Therefore, we believe that it is important to formulate an action of prevention, that should be implemented in all those areas at greater risk of contracting the infection.

Related to this, our study highlighted an interesting data, that children are infected mainly inside familial clusters [19, 20], while to a lesser extent at school or during sports activities. This finding is important and draw attention the importance of parental vaccination in limiting the spread of SARS-CoV-2.

Analyzing the children’s characteristics and clinical features of infection, our data are in line with those already present in the literature; in fact, in our children we found that the most frequent blood groups were group A and 0 Rh-positive, because seems that subjects with blood group A are more susceptible to infection [21, 22], while the group 0 is the most common in the Italian population. In addition, 27.5% of our children were asymptomatic or exhibit mild symptoms (72.5%) and could be treated at home in 99% of cases [6, 8, 23], requiring hospitalization in only 1% of cases.

While, related to comorbidities, we found that 15,3% of our infected children were allergic subjects; however, the role of atopy as risk factor for COVID-19 is very debated [24–27]

In children who performed serologic tests for anti-SARS-CoV-2 IgM and IgG 3 months after infection, we observed a negative response in a small percentage of patients (16.7% of cases). These data confirm our previous study in which we observed that antibody responses against SARS-CoV-2 can wane also in pediatric age [10], suggesting other possible mechanism underlying immunological memory. In line with this concept none of our children, who experienced a new contact with an infected subject, then tested positive to the control swab/then developed further episodes of COVID-19.

Furthermore, we found that children, like the adult population [15, 16, 28], may also experience persistent symptoms but with a lower prevalence. In our report 20% experienced persistent symptoms after the primary infection. This percentage is lower than that described by Buonsenso et al. [18], moreover the most common symptoms referred (fatigue, insomnia, difficulty in concentration and memory, muscle and joint pain, respiratory problems and palpitations), are in line with those described in other pediatric reports [17, 18, 29, 30].

Interestingly, persistent symptoms occurred predominantly in children with a longer duration of SARS-CoV-2 infection, older in age, and with a higher body mass index.

The older age as a risk factor for persistent symptoms after SARS-CoV-2 infection in children has already been demonstrated in other studies [31–33].

Other risk factors described for persistent symptoms after infection include: allergic diseases [31], muscle pain on admission and intensive care unit admission [33].

Related to a higher body mass index, it is known that overweight is characterized by a condition of chronic inflammation [34] and reduced immune function [35, 36] that could therefore predispose to the development of the long COVID syndrome. In fact, the proposed pathophysiological mechanisms underlying long COVID remain unclear and include: hyperinflammatory state, inadequate immune response, virus persistence and organ damage [37].

Overall, our data confirm the risk of persistent symptoms after infection even in pediatric age and highlight the need for a multidisciplinary follow-up, regardless of the severity of the initial symptoms, also in view of recent evidence of possible organ damage induced by the inflammatory process in different areas: brain [38], lung [39], heart [40].

Some of the data from our survey on the analysis of lifestyle changes and psychological impact during the period of illness are worrying. In particular, we showed that during the period of illness the most time spent by children was in front of the tv-video and social media and with phone cell. This effect was particularly evident in middle and high school children. These data need to be evaluated carefully, considering the negative effects related to prolonged use of electronic media on children. In fact, these negative effects concern not only the cognitive aspects, as difficulties with language acquisition, learning and poorer school results [41, 42] but especially physical effect [43–45], as the relationships between screen media exposure and the increased risks of overweight /obesity in pediatric age. In fact, we found that about 30% of children interviewed presented an increase in body weight compared to the period prior to the illness and only 14% of children practiced physical activity.

On the other hand, although the hours spent studying showed an upward trend among age groups, the average number of hours was about 3.5, which is low when considering the hours that a student normally spends studying between school and home; this consideration is even more worrisome if we look at the prolonged periods of lockdown, periods of illness related to the first infection and periods of convalescence related to long covid symptomatology..

In fact, our survey showed that more than half of parents believe that this type of teaching has a negative influence on their child's learning and educational growth. In favor of the re-opening of schools, we must consider what we have pointed out, in agreement with Munro et al. [46], that school is not one of the most dangerous places for the spread of coronavirus. We think that it is important to ensure school attendance in presence, not only because the school represents a place of education, of passing on knowledge and notions, but especially for the psychophysical and relational development of children and adolescents. Not surprisingly, the same children in our survey reported that attending school was the second most missed activity.

Furthermore, school closures could have a long-term negative economic impact, as well as amplify and worsen gender and social class inequalities to the disadvantage of the poorest (in terms of access to food with the development of severe nutritional deficiencies or inability to follow distance learning) and children with severe cognitive and physical disabilities; in addition, spending more time at home in a condition of severe socioeconomic stress could lead to an increase in cases of child abuse, as already described during this pandemic [47, 48].

Certainly, we think that the return of children to school or other extracurricular activities in person should be done safely, through the implementation of prevention strategies such as distancing, hand washing and use of the mask even in pediatric age [49–51]

This could have many advantages in pediatric care, as the significant reduction of pediatric infectious diseases disseminated through droplet and contact with the consequent drastic decrease of admissions, outpatient visits and emergency room accesses, as observed from the onset of pandemic with the diffusion of containment measures [52–55].

This study has some limitations: primarily, we have considered all persistent symptoms after resolution of infection, regardless of distance from infection although the definition of “long covid” is still much debated; secondary we also acknowledge that symptoms were only reported through online survey rather than directly ascertained and are related to a single-centre of study.

## Conclusions

We believe it is important to highlight from our survey that children at risk for persistent symptoms after infection are primarily those who are older, have higher weight, and have a longer duration of primary infection. In particular, increased body weight associated with greater susceptibility to infection and longer duration illnesses, that promote sedentary activities, are associated with greater psychological impact of COVID-19, and these could be among the main causes of increased susceptibility of some children to developpersistent symptoms after SARS-CoV-2 infection.

## Data Availability

The datasets used and/or analysed during the current study are available from the corresponding author on reasonable request.

## Abbreviations

COVID-19: Coronavirus disease 2019
ASL of Latina: Latina Local Health Authority
MIS-C: Multisystem Inflammatory Syndrome
LCC: Long covid children
HC: Healthy children
